# Caffeic Acid Phenylethyl Amide Protects against the Metabolic Consequences in Diabetes Mellitus Induced by Diet and Streptozocin

**DOI:** 10.1155/2012/984780

**Published:** 2012-06-24

**Authors:** Yi-Chun Weng, Sung-Ting Chuang, Yen-Chu Lin, Cheng-Fung Chuang, Tzong-Cherng Chi, Hsi-Lin Chiu, Yueh-Hsiung Kuo, Ming-Jai Su

**Affiliations:** ^1^Institute of Pharmacology, College of Medicine, National Taiwan University, Taipei 10051, Taiwan; ^2^Graduate Institute of Medical Sciences, Chang Jung Christian University, Tainan 71101, Taiwan; ^3^Tsuzuki Institute for Traditional Medicine, China Medical University, Taichung 40402, Taiwan

## Abstract

Caffeic acid phenyl ester is distributed wildly in nature and has antidiabetic and cardiovascular protective effects. However, rapid decomposition by esterase leads to its low bioavailability in vivo. In this study, chronic metabolic and cardiovascular effects of oral caffeic acid phenylethyl amide, whose structure is similar to caffeic acid phenyl ester and resveratrol, were investigated in ICR mice. We found that caffeic acid phenylethyl amide protected against diet or streptozocin-induced metabolic changes increased coronary flow and decreased infarct size after global ischemia-reperfusion in Langendorff perfused heart. Further study indicated that at least two pathways might be involved in such beneficial effects: the induction of the antioxidant protein MnSOD and the decrease of the proinflammatory cytokine TNF**α** and NF**κ**B in the liver. However, the detailed mechanisms of caffeic acid phenylethyl amide need further studies. In summary, this study demonstrated the protective potential of chronic treatment of caffeic acid phenylethyl amide against the metabolic consequences in diabetes mellitus.

## 1. Introduction

Diabetes mellitus (DM) is a metabolic disease with hyperglycemia and usually accompanied with many complications [1–5]. Lifestyle patterns in industrialized societies comprise an increasing availability and ingestion of high-caloric food in the presence of a sedentary living, and these factors are emerging as the fundamental causes of the fast-spread diabetes. Since the incidence of acute myocardial infarction and coronary heart disease is pretty high in the population of metabolic syndrome, the identification of new pharmacological approaches to effectively prevent and treat metabolic syndrome and its cardiovascular complications is of crucial importance.

Streptozocin (STZ) is a pancreatic *β*-cell toxin that induces rapid and irreversible necrosis of *β* cells. It is widely used in making experimental animal models of type 1 DM [6]. Since insulin secretion is deficient in STZ-induced type 1 diabetic mice, it is also a good model for research on insulin-independent antidiabetic mechanisms of the compounds. For type 2 DM, transgenic [7, 8] and chemical-induced [9] animals are wildly used in hypoglycemic drug screen for many years, but these animals are not so similar to most clinical patients. Recently, the usage of diet-induced type 2 DM animal models in studies has increased [10–15]. Higher similarity in the cause and the pathology of DM in these diet-induced animal models are observed as compared to those in patients. According to our previous results, two stages were observed in high-fat and high-fructose diet-induced diabetic mice: hyperglycemia and hyperlipidemia without insulin resistance occurred at week 2 and systemic insulin resistance owning to low insulin sensitivity in main metabolic tissues occurred at week 4 [16]. Since systemic insulin resistance and specific reduction of insulin sensitivity in major metabolic tissue could be induced in a short time, diet-induced diabetic BLTW : CD1(ICR) mice could be an efficient model for medical research with an advantage of ruling out strain-specific gloss.

**Figure 1:**
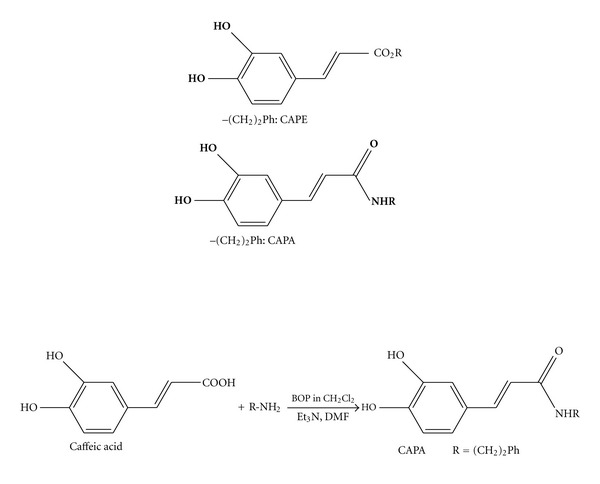
The chemical structures of CAPE and CAPA used in the present study. CAPA was obtained from the amide binding coupling method, beginning with caffeic acid.

Many natural polyphenolic compounds are demonstrated to have anti-inflammatory, antioxidant, anticarcinogenic, antithrombotic, and cardiovascular protective effects [17–19]. Resveratrol and curcumin are successfully employed in the prevention and treatment of a variety of diseases, including metabolic dysfunction, coronary artery disease, pressure-overload hypertrophy, and heart failure [20, 21]. Caffeic acid and caffeic acid phenyl ester (CAPE) are also widely distributed in nature, especially the plant kingdom. However, the rapid metabolism of CAPE by esterase leads to its low bioavailability. Caffeic acid phenylethyl amide (CAPA) was a caffeic acid amide derivative and structurally similar to CAPE and resveratrol (Figure 1). Since amide is more resistant to esterase, it is foreseeable that CAPA is more stable than CAPE in vivo. In this study, the protective potential of chronic oral CAPA against the metabolic consequences in type 1 and type 2 diabetic mice model was investigated and the known antidiabetic agent metformin was taken as a positive control.

## 2. Materials and Methods

### 2.1. Chemical

Beginning with caffeic acid, CAPA was obtained from the following amide binding coupling method. The solution of benzotriazol-1-yloxytris (dimethylamino)phosphonium hexafluorophosphate (BOP) (1.2 eq) in dichloromethane (CH_2_Cl_2_) (5 mL) was added to a mixture of caffeic acid (100 mg), R-NH_2_ (1.2 eq), and triethylamine (Et_3_N) (0.08 mL) in dimethylformamide (DMF) (1.0 mL). The mixture was stirred at 0°C for 30 min, then allowed to stir at room temperature for 12 h. This reaction mixture was evaporated under in vacuo, and the residue was partitioned between ethyl acetate (AcOEt) and H_2_O. Successively, the AcOEt layer was washed with 3 N aqueous HCl and 10% NaHCO_3_(aq), dried over MgSO_4_ and concentrated in vacuo. The residue was further purified by column chromatography with eluting solution (CH_2_Cl_2_–AcOEt 1 : 1, v/v) on silica gel (70–230 and 230–400 mesh, Merck 7734). The final products (82–88% yield) were recrystallized from AcOEt to obtain pure crystals. ^1^H and ^13^C NMR spectra were recorded on a Bruker Avance 500 spectrometer. Electron impact mass spectrometries (EIMS) were determined on a Finnigan TSQ-46C mass spectrometer. IR spectra were recorded on a Nicolet Magna-IR 550 spectrophotometer.

Caffeic acid phenylethyl amide: solid. mp 148-149°C. IR *ν*_max⁡_ (cm^−1^): 3288, 1642, 1591, 1523, 1361, 1279, 1036, 975, 849. ^1^H NMR (CD_3_COCD_3_, 500 MHz): **δ** 2.84 (2H, *t*, *J* = 6.8 Hz), 3.53 (2H, *q*, *J* = 6.8 Hz), 6.43 (1H, *d*, *J* = 15.2 Hz), 6.83 (1H, *d*, *J* = 8.1 Hz), 6.92 (1H, *dd*, *J* = 8.1, 1.8 Hz), 7.07 (1H, *d*, *J* = 1.8 Hz), 7.15–7.30 (5H, *m*), 7.35 (1H, *br*. *s*, –NH), 7.43 (1H, *d*, *J* = 15.2 Hz), 8.20 (1H, *s*, –OH), 8.42 (1H, *s*, –OH). EI-MS *m/z*(%): 283 (M^+^, 17), 178 (22), 163 (100).

### 2.2. Animals

4-week-old male BLTW: CD1(ICR) mice were acquired from BioLasco Taiwan Co., Ltd. and maintained at National Taiwan University College of Medicine Experimental Animal Center, in a temperature- and humidity-controlled (22±1°C and 60 ± 5%) environment with a strict 12 hour light-dark cycle and given free access to food and water. After the acclimatizing period (at least 3 days), mice with fasting plasma glucose levels higher than 130 mg/dL or lower than 70 mg/dL were excluded.

Type 1 diabetic mice were induced by modifying the previous method [6]. In brief, an intraperitoneal injection of streptozocin (STZ, Sigma Chemical Co.; St. Louis, MO, USA) at 150 mg/kg dissolved in 1% citrate buffer was performed in 4-week-old mice fasted for 48 hours. Mice with plasma glucose level of 350 mg/dL or greater were considered as type 1 diabetic. Type 2 diabetic mice were induced by high-fat and high-fructose diet according to previous method [22] and our previous study [16]. Mice with fasting plasma glucose level of 150 mg/dL or greater were considered as type 2 diabetic.

Caffeic acid phenylethyl amide (10 mg/kg) or metformin (300 mg/kg) was given once a day orally. The investigation followed the University guidelines for the use of animals in experimental studies and conformed to the Guide for the Care and Use of Laboratory Animals published by the US National Institutes of Health (NIH publication no. 85-23, revised 1996). The animal experiments were approved by the IACUC of National Taiwan University (IACUC no. 20100007).

### 2.3. High-Fat and High-Fructose Diet

 Additional 24.18% w/w palm oil and 2.56% w/w soybean oil were added to powder of Purina Laboratory Rodent Diet 5001 standard chow (Purina; PMI Nutrition International, St Louis, MO, USA) and this high-fat diet mixture was reconstituted in small pellets. High fat diet and fructose-sweetened water (containing 20% fructose) were used to induce type 2 diabetes in ICR mice. Metabolizable energy of standard rodent chow, HF diet, and fructose water was 3.04 kcal/gm, 4.63 kcal/gm, and 0.8 kcal/mL, respectively.

### 2.4. Blood Sampling

Mice were anesthetized with pentobarbital (80 mg/kg, intraperitoneal, Sigma), and blood was withdrawn from the orbital venous plexus using a heparinized capillary tube. Blood samples were centrifuged at 10000 xg for 5 min, and plasmas were placed on ice or stored at −20°C until assay [23].

### 2.5. Determination of Plasma Parameters

An aliquot of plasma was added to glucose kit reagent (Biosystems S.A., Barcelona, Spain) and incubated at 37°C for 5 min. The concentration of plasma glucose was then estimated via a spectrophotometer with samples run in duplicate [24]. Determination of serum insulin concentration adopted ELISA (Mammalian Insulin ELISA; Mercodia AB, Uppsala, Sweden) [25]. Plasma triglycerides and total cholesterol were measured using commercially available cholesterol kit (Randox, UK) and triglycerides kit (Randox, UK), respectively. Plasma retinol binding protein 4 (RBP4) and adiponectin levels were measured using commercially available ELISA kits (AdipoGen and Linco, resp.). All assays were performed according to the manufacturer's instructions.

### 2.6. Insulin Tolerance Test (ITT) and Intraperitoneal Glucose Tolerance Test (IPGTT)

Intraperitoneal glucose tolerance test (IPGTT) and insulin tolerance test (ITT) were performed according to the method described previously [12, 26]. Briefly, intraperitoneal insulin (0.5 IU/kg) and glucose (2 g/kg) tolerance tests were performed after 3 hr and overnight of fast, respectively, and blood samples were collected at 0, 15, 30, and 60 minute for ITT and at 0, 30, 60, 120, and 150 minute for IPGTT. Areas between glucose curves after glucose or insulin injection and baseline glucose level curve (ΔAUC) were also calculated.

### 2.7. Glycogen Content Assay

About 50 mg of tissue sample was dissolved in 1 N KOH at 70°C for 30 min. Dissolved homogenate was neutralized by glacial acetic acid and incubated overnight in acetate buffer (0.3 M sodium acetate, pH 4.8) containing amyloglucosidase (Sigma, St. Louis, MO, USA). Samples were then analyzed by measuring glucosyl units using Trinder reaction. The reaction mixture was neutralized with 1 N NaOH [27].

### 2.8. Histological Analysis

Tissues were immersion-fixed in neutral 10% buffered formalin. Sections were paraffin-embedded, cut at 4 *μ*m, and mounted onto slides.HEstaining was performed for histological analysis. Tissue sections were examined using a microscope and were photographed with a digital camera.

### 2.9. Cell Culture

 HepG2 cell lines were grown, as a monolayer, in Dulbecco's modified Eagle's medium (DMEM) supplemented with 10% (v/v) fetal bovine serum (FBS), 100 U/mL penicillin G, and 100 *μ*g/mL streptomycin in a humidified atmosphere with 5% CO_2_ at 37°C. The cells were cultured at 90% confluence and then seeded onto 6 cm culture dishes to perform the experiments [28]. Cells were starved for 24 h before the experiments. After starvation, cells were incubated in DMEM medium containing 10% FBS and treated with various concentrations of caffeic acid phenylethyl amide at 1 h prior to and during treatment with human recombinant TNF-*α* (tumor necrosis factor-alpha, CytoLab Ltd., Rehovot, Israel) or H_2_O_2_ (100 to 500 *μ*M). Incubation with TNF-*α* was conducted for 60 min for IKK (I*κ*B kinase) phosphorylation as described by others [29].

### 2.10. HepG2 Triglyceride Content Assay

Cells were lysed in lysis buffer (20 mM HEPES (pH 7.6), 150 mM NaCl, 1% Triton X-100, 0.1% SDS) and total fat was extracted by Bligh and Dyer method [28]. Briefly, the cell extract was incubated with methanol and chloroform for 1 h, and then chloroform and sterile water were added, centrifuged briefly to collect chloroform phase. This extract was dried for overnight, and was dissolved in 10% Triton-isopropanol solution. According to a manual of triglycerides kit, the quantity of triglyceride was measured.

### 2.11. Western Blotting

Protein contents were measured by Western blotting using commercially available polyclonal antibodies specific for TNF*α* (Millipore, USA), MnSOD (Millipore, USA), p-p65 (Cell Signaling, USA), p-IKK*α*/*β* (Santa Cruz, USA), PEPCK (a kind gift from Professor DK Granner), and GLUT4 (R&D systems). To adjust for loading differences, blots were reprobed with a monoclonal antibody to *β*-actin (ABS, USA). Densities of the obtained immunoblots were quantified using ImageQuant.

### 2.12. Langendorff Perfused Heart Model

Isolated perfused mouse heart model was set as previously described with minor modification [30]. In brief, the aorta was cannulated with a 20-gauge cannula. The aorta was perfused in the Langendorff System (ADInstruments) at constant pressure (80 ± 3 mmHg). The hearts were perfused with a buffer consisting of (in mM) 118.5 NaCl, 25 NaHCO_3_, 4.7 KCl, 1.2 MgSO_4_, 1.2 KH_2_PO_4_, and 2.5 CaCl_2_, 11 glucose and gassed with 95% O_2_-5% CO_2_ (pH 7.4). Hearts were allowed to beat spontaneously; basal coronary flow and heart rate were recorded after a 30-minute balance period. Then hearts were subjected to a 30-minute global ischemia (area at risk equaled to whole heart mass) and 2-hour reperfusion. The infarct sizes were analyzed by TTC stain method and expressed as percentage of AAR.

### 2.13. Statistical Analysis

Data were represented as the means±SEM for the number (*n*) of animals in the group as indicated in figures. Statistical analysis was performed by one-way analysis of variance (ANOVA) with Dunnett's post-hoc test. *P* < 0.05 was regarded as statistically significant.

## 3. Results

### 3.1. CAPA Protected Mice from Diet-Induced Metabolic Dysfunction

**Figure 2:**
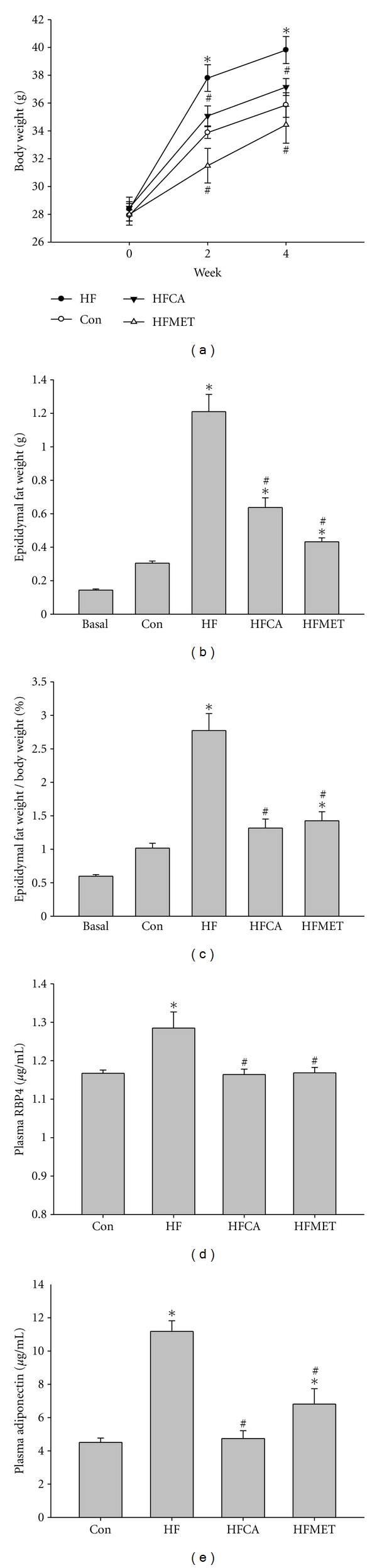
Fat metabolism of mice. (a) Body weight before (0W) and after two (2W) and four (4W) weeks of diet exposure. (b and c) Fat mass and fat to body weight ratio after 4 weeks of diet exposure. (d and e) Plasma levels of retinol binding protein 4 (RBP4) and adiponectin after 4 weeks of diet exposure. *n* = 6–8 per group; means ± SEM; Con: control mice; HF: mice fed with high fat and diet; HFCA: HF mice treated with CAPA; HFMET: HF mice treated with metformin; *: *P* < 0.05 versus Con; #: *P* < 0.05 versus HF.

Continuous exposure to a high-fat and high-fructose diet for 4 weeks led to significant increases in body weight (Figure 2(a)) and fat mass (Figures 2(b) and 2(c)) in ICR mice. Significant lower body weight and fat mass accompanied by decreased calorie intake (Table 1) were observed in mice treated with 10 mg/kg CAPA or 300 mg/kg metformin compared with nontreated ones. Plasma retinol binding protein 4 (RBP4) and adiponectin levels were increased after exposure to the high-fat and high-fructose diet, and both levels were lowered after CAPA treatment (Figures 2(d) and 2(e)).

### 3.2. CAPA Protected Mice from Glucose Intolerance

**Figure 3:**
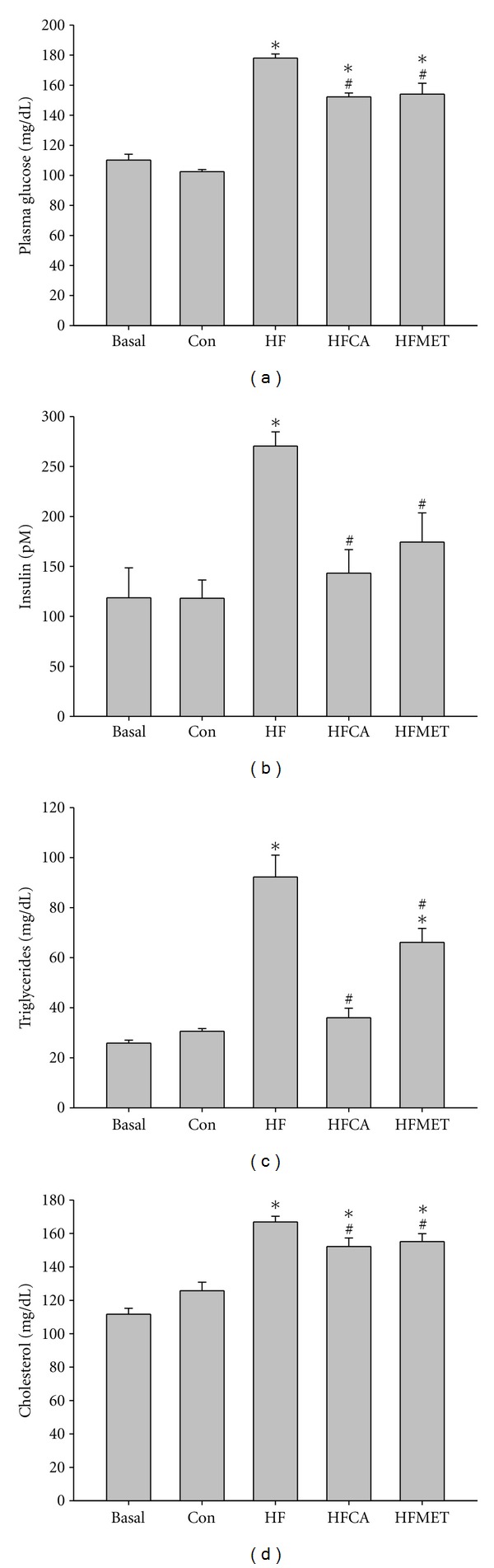
Diet-induced hyperglycemia and hyperlipidemia were reduced by CAPA treatment in BLTW : CD1(ICR) mice. (a) Plasma glucose levels after 4 weeks of diet exposure. (b) Plasma insulin levels after 4 weeks of diet exposure. (c) Plasma triglycerides levels after 4 weeks of diet exposure. (d) Plasma total cholesterol levels after 4 weeks of diet exposure. *n* = 6–8 per group; means ± SEM; Con: control mice; HF: mice fed with high fat and diet; HFCA: HF mice treated with CAPA; HFMET: HF mice treated with metformin; *: *P* < 0.05 versus Con; #: *P* < 0.05 versus HF.

**Figure 4:**
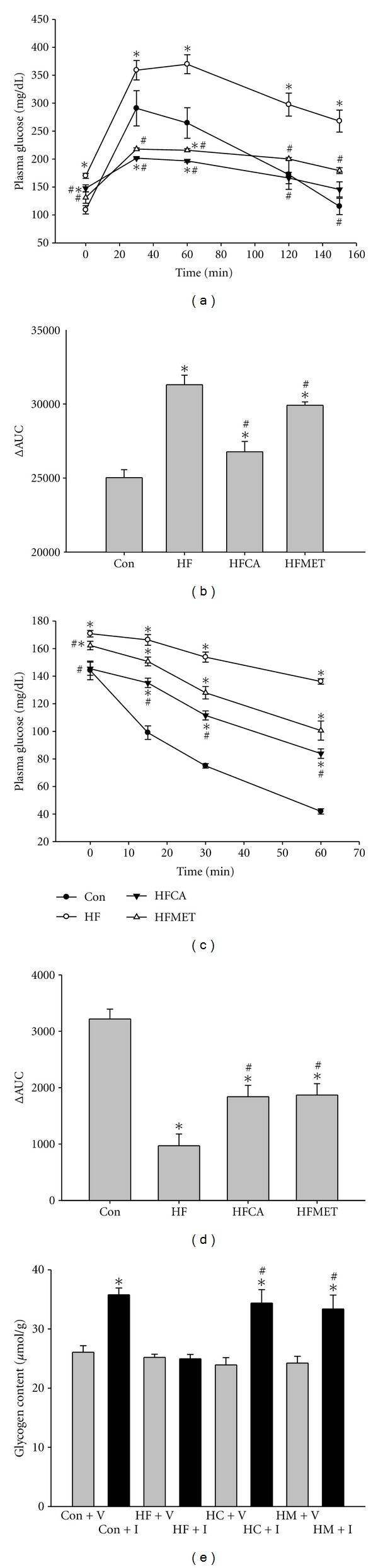
BLTW : CD1(ICR) mice are protected from diet-induced glucose intolerance. (a) Glucose tolerance test (GTT) after 4 weeks of diet exposure by using an i.p. dose of 2 g of glucose per kg of body weight. (b) Values of the area under the curve (AUC) during GTT since the different basal plasma glucose levels, areas between glucose curves after glucose or insulin injection, and baseline glucose level curve (ΔAUC) were calculated. (c) Insulin tolerance test (ITT) after 4 weeks of diet exposure by using 0.5 IU/kg insulin. (d) ΔAUC during ITT. *n* = 6–8 per group; means ± SEM; *: *P* < 0.05 versus Con; #: *P* < 0.05 versus HF. (e) Glycogen content assay after 4 weeks of diet exposure by using 0.5 IU/kg insulin. *n* = 6–8 per group; means ± SEM; *: *P* < 0.05 versus those treated with vehicle (V, grey bars); #: *P* < 0.05 versus HF treated with insulin (I, black bars); Con: control mice; HF: mice fed with high fat and diet; HFCA or HC: HF mice treated with CAPA; HFMET or HM: HF mice treated with metformin.

Chronic exposure to high-fat and high-fructose diet for 4 weeks increased glucose, insulin, triglyceride, and cholesterol levels (Figure 3(a) to 3(d)). To assess the impact of chronic high-fat and high-fructose diet exposure on glucose homeostasis in more detail, the mice were subjected to a glucose tolerance test (GTT) (Figure 4(a)). Intraperitoneal administration of glucose led to a more rapid increase of blood glucose levels in mice fed a high-fat and high-fructose diet, indicating the expected diet-induced systemic glucose intolerance. Both CAPA and metformin treated mice had lower blood glucose levels than nontreated mice on the same glucose tolerance test. ΔAUC (area-under-the-curve) values revealed a significantly preserved glucose tolerance of mice treated with CAPA (Figure 4(b)). An insulin tolerance test (ITT) after chronic exposure to high-fat and high-fructose diet for 4 weeks showed lower glucose levels and bigger ΔAUC values in CAPA and metformin treated mice (Figures 4(c) and 4(d)). Glycogen content assay also showed that CAPA and metformin preserved the insulin induced glycogen synthesis in skeletal muscle (Figure 4(e)).

Noteworthy, even when hyperglycemia, hyperinsulinemia, and higher plasma level of adiponectin had been developed after two weeks of exposure to high-fat and high-fructose diet, treatment with CAPA for another 2 weeks significantly ameliorated the metabolic dysfunctions and prevented glucose intolerance (Table 2). Similarly, mice with plasma glucose levels higher than 350 mg/dL measured at two weeks after STZ injection were seen as type 1 diabetic mellitus model. CAPA treatment for another two weeks ameliorated STZ-induced hyperglycemia and body weight (Table 3). According to these results, we concluded that chronic oral administration of CAPA could protect mice from diet and STZ-induced metabolic dysfunctions, even when hyperglycemia had been developed.

### 3.3. CAPA Preserved Basal Coronary Flow and Decreased Infarct Size in Langendorff Perfused Heart

Since the incidence of acute myocardial infarction and coronary heart disease is pretty high in the population of metabolic syndrome, we investigated the effects of 2-week treatment with CAPA or metformin on basal coronary flow and infarct size after global ischemia-reperfusion in Langendorff perfused heart. Four weeks of continuous exposure to high-fat and high-fructose diet decreased basal coronary flow, but CAPA or metformin treatment (introduced after 2 weeks exposure to the diet) for two weeks preserved the basal flow (Table 2). After a 30-minute global ischemia and 2-hour reperfusion, mice fed on high-fat and high-fructose diet had larger infarct size. Treatment of CAPA or metformin protected mice on high-fat and high-fructose diet from severe ischemic and reperfusion injury (Table 2). Besides, CAPA treatment could also improve basal coronary flow and decreased infarct size in STZ-induced type 1 diabetic mice (Table 3).

### 3.4. CAPA Protected Liver from Inflammation and Decreased Gluconeogenesis

**Figure 5:**
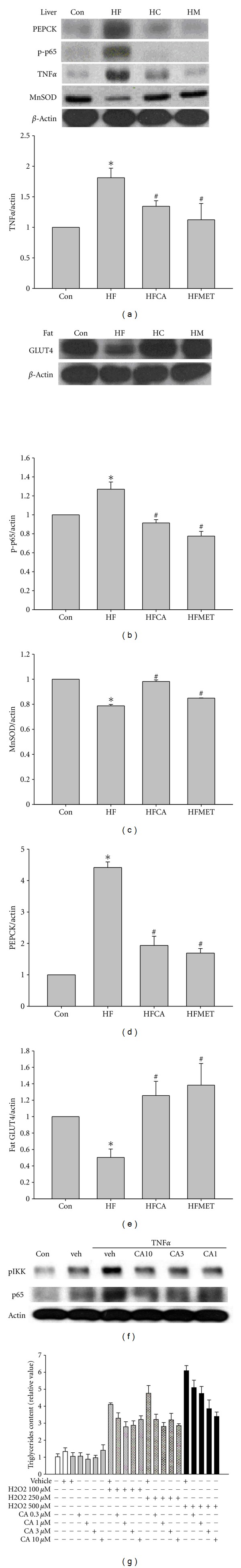
Protein expressions in the livers and fats of BLTW : CD1(ICR) mice after 4 weeks of diet exposure and triglycerides accumulation in HepG2 cells. (a) Protein expression of TNF*α* in livers of mice. (b) Protein expression of p-p65 in livers of mice. (c) Expression of the antioxidant protein MnSOD in livers of mice. (d) Expression of PEPCK in livers of mice. (e) Expression of GLUT4 in fats of mice. (f) Protein expression of pIKK*α*/*β* and p-p65 in HepG2 cells. (g) H_2_O_2_ induced triglycerides accumulation in HepG2 cells. *n* = 3 per group; means ± SEM; Con: control mice; HF: mice fed with high fat and diet; HFCA or HC: HF mice treated with CAPA (CA); HFMET or HM: HF mice treated with metformin; *: *P* < 0.05 versus Con; #: *P* < 0.05 versus HF.

A high-fat and high-fructose diet-induced obesity can induce and lead to a chronic inflammatory reaction, which is believed to be critical for the development of glucose intolerance and insulin resistance [31]. Correspondingly, the expression of TNF*α*, a major proinflammatory cytokine, was significantly enhanced in livers of mice on a chronic high-fat and high-fructose diet (Figure 5(a)). However, CAPA or metformin treated mice exhibited lower levels of TNF*α*. Consistent with the inhibition of TNF*α* expression, both CAPA and metformin treatment resulted in lower p-p65 expression (Figure 5(b)). In contrast to the suppression of TNF*α* expression, the antioxidant proteins manganese superoxide dismutase (MnSOD) expression was increased in CAPA or metformin treated mice on high-fat and high-fructose diet (Figure 5(c)).

RBP4 is known as a key regulator in obesity-related insulin resistance and type 2 DM. RBP4 secreted from adipocytes is increased when GLUT4 is downregulated. PEPCK is an important protein for gluconeogenesis and RBP4 increases gluconeogenesis in liver via upregulating PEPCK and inhibit insulin signaling [32]. After treatment of CAPA or metformin for four weeks, expression of PEPCK in the livers decreased and expression of GLUT4 in the fats increased (Figures 5(d) and 5(e)).

In summary, treatment with CAPA appears to protect mice from high-fat and high-fructose diet-induced hepatic inflammation and glucose intolerance associated with decreasing the NF*κ*B-mediated induction of inflammatory cytokines and increasing the expression of antioxidant protein. We used HepG2 as cell models for further investigation. Incubation with TNF*α* increased pIKK*α*/*β* expression and NF*κ*B activation in HepG2, and cotreatment with CAPA could ameliorate TNF*α* induced pIKK*α*/*β* expression (Figure 5(f)). In addition, we analyzed triglyceride content in HepG2 cells treated with H_2_O_2_. Cells were stimulated with H_2_O_2_ (100, 250, and 500 *μ*M) for 3 hours and then cultured for another 48 hours in fresh DMEM without H_2_O_2_ for triglyceride assay. The triglyceride levels rose with increasing H_2_O_2_ concentrations. Incubation with CAPA (1, 3, and 10 *μ*M) 1 h before H_2_O_2_ prevent triglycerides accumulation in HepG2 cells (Figure 5(g)).

## 4. Discussion and Conclusion

Our study addresses the potential of CAPA in arresting the development of glucose intolerance and cardiac ischemic injury in BLTW : CD1(ICR) mice. Our data show that CAPA improves lipid and glucose metabolism and basal coronary flow and protects myocardium from damage inflicted by high-fat and high-fructose diet or STZ. The induction of the antioxidant protein MnSOD and the decrease of NF*κ*B activation in the liver were also observed after CAPA treatment. Since CAPA also decreased plasma glucose levels and protected cardiac ischemic injury in insulin deficient STZ mice, insulin-independent mechanisms might be involved.

White adipose tissue is now recognized as a secretary organ and secrets adipocytokines, such as RBP4 and adiponectin, which are involved in the regulation of glucose and lipids metabolism. Though it is reported that plasma adiponectin level is lower in obese, insulin resistant, or diabetic patients, in this study we found that high fat and fructose diet did not cause lower plasma adiponectin level. Liu et al. [33] observed that plasma adiponectin levels were higher when wild type mice were fed with high-fat diet less than 12 weeks. However, plasma adiponectin levels decreased after wild type mice were fed with high-fat diet longer than 12 weeks. Our finding is consistent with Liu's report that mice fed with high-fat diet for two and four weeks had higher plasma adiponectin levels than control mice and might lead to the conclusion that a low plasma adiponectin level might be a result instead of a cause in insulin resistance. Liu et al. also showed that compared with wild type, adiponectin receptor 2 deficient mice always had higher plasma adiponectin levels and suffered less from diet-induced insulin resistance, yet deteriorated glucose homeostasis as high-fat feeding continued, which resulted from the failure of pancreatic beta-cells to adequately compensate for the moderate insulin resistance. However, we could not tell whether adiponectin receptors are involved in diet-induced insulin resistance in our study yet. Another limitation is that we did not analyze high molecular weight adiponectin (HMW adiponectin). A decrease level of HMW adiponectin is a predictor of progression to metabolic syndrome. Lifestyle modification for three months or administrating thiazolidinediones induced an increase in serum HMW adiponectin in patients with metabolic syndrome [34]. We did not know how plasma levels of HMW adiponectin changed after high-fat diet exposure or CAPA treatment in this study.

Yang et al. [35] showed that RBP4 secretion from adipocytes is increased when GLUT4 is downregulated. In this study, increased RBP4 secretion and decreased GLUT4 expression in fat were observed after 4 weeks exposure to the high-fat and high-fructose diet, confirming that insulin resistance occurred in adipose tissue of mice fed with high-fat and high-fructose diet. Obvious increased PEPCK expression in liver, an important index of insulin resistance, was observed in HF mice. Chronic treatment of CAPA and metformin significantly decreased body weight gain, fat mass and plasma levels of adipocytokines induced by high-fat and high-fructose. These data indicated that insulin resistance had occurred in liver of mice after four weeks of high fat and high fructose diet feeding and oral CAPA (10 mg/kg) exerted similar protection in mice against these metabolic dysfunctions as metformin (300 mg/kg) did.

However, it is noteworthy that CAPA preserved MnSOD in the liver at a higher level than metformin did (*P* < 0.05). Since oxidative stress is reported to induce triglycerides accumulation in hepatocytes and might play important role in hepatic insulin resistance [28], we stimulated HEPG2 with H_2_O_2_ and found that CAPA decreased the triglycerides accumulation in HEPG2 after H_2_O_2_ stimulation. This observation is correlated with the prominent reduction of plasma triglycerides by CAPA in diet-induced diabetic mice. However, whether induction of MnSOD was required in this effect of CAPA needs further studies.

Hepatic inflammation is an established risk factor for the development of insulin resistance and glucose intolerance [36]. Insulin resistance and glucose intolerance, in turn, can stimulate the development and progression of hepatic inflammation and hepatosteatosis [37], thereby fueling and promoting a detrimental cycle that is believed to represent a key role in metabolic syndrome. In this study, daily oral CAPA results in protection of mice from diet-induced glucose intolerance. Hepatic NF*κ*B signaling is activated by high-fat and high-fructose diet exposure and triggers insulin resistance [38], thereby linking inflammation with obesity-induced insulin resistance [39]. Conjunction with the activation of antioxidant protein MnSOD, inhibition of NF*κ*B by CAPA may explain the prevention from hepatic inflammation and diet-induced glucose intolerance.

Most importantly, clinical diabetes mellitus is usually diagnosed when hyperglycemia occurs; thus, it is important to find out antidiabetic compounds that can treat, not prevent, metabolic dysfunctions after hyperglycemia has occurred. CAPA can arrest the development of diabetes mellitus and the metabolic consequences in type 1 and type 2 diabetic mice model even when hyperglycemia has developed. After blood glucose levels were raised, daily oral CAPA for two weeks prevented body weight gain and hyperglycemia and preserved basal coronary flow in Langendorff perfused hearts. When subjected to global ischemia and reperfusion, larger infarct sizes were observed in the hearts from type 1 and type 2 diabetic mice. Chronic oral CAPA prevented severe ischemic injury in diabetic hearts.

In summary, chronic oral CAPA protects against diet-induced metabolic damage, such as obesity, hyperglycemia, insulin resistance and liver inflammation. CAPA also increased coronary flow and decreased infarct size after global ischemia-reperfusion in Langendorff perfused heart. In addition, CAPA protects mice from metabolic consequences by STZ-induced type 1 diabetes, indicating that some effects of CAPA were insulin independent. Prevention of activation of the proinflammatory NF*κ*B pathway and increase in expression of antioxidant proteins such as MnSOD in the liver were also involved in the action of CAPA. Together, these results highlighted the protective potential of chronic treatment of CAPA against the metabolic consequences in diabetes mellitus.

However, there are still lots of work to do. Whether decreased calorie intake contributed to these beneficial metabolic effects after CAPA treatment and whether CAPA protected cardiovascular system directly were still left unclarified. Changes were still taking place and steady state was not reached after four weeks of feeding. Thus, short study period might be a limitation in our present study. A long study period, such as 12 weeks or longer, should be taken to estimate the long-term effects of CAPA in ICR mice. Moderate Sirt1 (mammalian silent information regulator 2) overexpression under control of its natural promoter in mice prevents high-fat diet-induced glucose intolerance, and this effect may result, in part, from prevention of high-fat diet-induced activation of the proinflammatory NF*κ*B pathway together with upregulation of PGC1*α* and its antioxidant targets such as MnSOD or Nrf1 [40]. Since CAPA is structurally similar to the Sirt1 activator, resveratrol, further study was needed to clarify whether Sirt1 activation was involved in the beneficial effects of CAPA. In addition, CAPA had AMPK (AMP-activated protein kinase) activating activities in C2C12 skeletal muscle cells and in HEP3B hepatocytes (our unpublished data), but further investigations should be done to know whether and how AMPK activation was involved in the protective effects of CAPA. Finally, the anti-inflammatory effects of CAPA open up aspects that could link caffeic acid phenylethyl amide with other diseases such as aging and cancer. Although the detailed mechanisms need to be clarified, the protective potential of chronic treatment of CAPA against diabetes mellitus and other diseases is undeniable.

## Figures and Tables

**Table 1:** Food and water intake of mice after 4 weeks of diet exposure.

	Basal	After 4 weeks of diet exposure			
		Con	HF	HC	HM
Body weight (g)	25.6 ± 0.4	35.5 ± 0.8	40.2 ± 1.0^∗^	37.2 ± 0.6^#^	35.0 ± 1.1^#^
Night food intake (g)	4.5 ± 0.5	6.0 ± 0.5	4.3 ± 0.8^∗^	2.4 ± 0.3^#^	2.4 ± 0.4^#^
Night water intake (mL)	10.5 ± 0.6	12.2 ± 0.2	6.4 ± 0.6^∗^	5.9 ± 0.3	6.4 ± 0.5
Night calorie intake					
Food (kcal)	14.4 ± 1.3	18.1 ± 1.4	19.8 ± 2.2	12.5 ± 0.9^#^	11.1 ± 1.7^#^
Water (kcal)	—	—	5.5 ± 0.3	4.1 ± 0.5	5.1 ± 0.4
Total (kcal)	14.4 ± 1.3	18.1 ± 1.4	25.3 ± 2.2^∗^	16.6 ± 0.8^#^	15.9 ± 4.4^#^

Con: control mice; HF: mice fed with high fat and diet; HC: HF mice treated with CAPA; HM: HF mice treated with metformin; ^∗^: *P* < 0.05 versus Con; ^#^: *P* < 0.05 versus HF; *n* *=* 6–8 per group.

**Table 2:** Metabolism and ischemic injury of mice after 4 weeks of diet exposure.

	After 4 weeks of diet exposure			
	Con	HF	HF2WCA2W	HF2WMET2W
Body weight (g)	35.5 ±* *0.8	40.2 ± 1.0^∗^	39.7 ± 1.2^∗^	38.3 ± 0.7^∗^
Plasma insulin (pM)	117.5* *±* *8.8	203.5* *±* *21.1^∗^	148.3 ± 29.2^#^	131.1 ± 20.5^#^
Plasma glucose during IPGTT (mg/dL)				
0 min after injection	109.2 ± 7.5	169.9 ± 4.5^∗^	149 ± 6.9^∗,#^	151.4 ± 8.5^∗, #^
30 min after injection	290.8 ± 31.5	359.0 ± 17.5^∗^	291.7 ± 2.4^∗, #^	271.8 ± 2.3^∗, #^
120 min after injection	172.2 ± 26.3	297.5 ± 20.5^∗^	166.7 ± 10.2^∗, #^	179.3 ± 5.2^∗, #^
Basal coronary flow (mL/min)	3.4 ± 0.5	1.6 ± 0.3^∗^	3.6 ± 0.4^#^	2.5 ± 0.4^#^
Infarct size of the heart after global ischemia/reperfusion (AAR%)	30.6 ± 5.1	39.4 ± 6.6^∗^	29.9 ± 4.8^#^	30.0 ± 3.8^#^

CAPA was introduced after 2 weeks of diet exposure and orally treated once a day for another 2 weeks. Con: control mice; HF: mice fed with high fat and diet; HF2WCA2W: HF mice treated with CAPA for another 2 weeks; HF2WMET2W: HF mice treated with metformin for another 2 weeks; AAR: area at risk; ^∗^: *P  *<* *0.05 versus Con; ^#^: *P  *<* *0.05 versus HF; *n* = 6–8 per group.

**Table 3:** Metabolism and ischemic injury of mice after 4 weeks of STZ injection.

	After 4 weeks of STZ injection		
	Con	STZ	S2WCA2W
Body weight (g)	35.5 ± 0.8	21.2 ± 2.9^∗^	24.0 ± 0.6^∗, #^
Plasma glucose (mg/dL)	113.5 ± 4.5	588.0 ± 34.5^∗^	483.2 ± 24.0^∗, #^
Plasma insulin (pM)	117.5 ± 8.8	8.3 ± 1.5^∗^	5.8 ± 1.0^∗^
Basal coronary flow (mL/min)	3.4 ± 0.5	1.5 ± 0.2^∗^	1.83 ± 0.3^∗, #^
Infarct size of the heart after global ischemia/reperfusion (% AAR)	30.6 ± 5.1	51.3 ± 2.5^∗^	37.1 ± 5.8^∗, #^

CAPA was introduced after 2 weeks of STZ injection and orally treated once a day for another 2 weeks. Con: control mice; STZ: streptozocin-induced type 1 diabetic mice; S2WCA2W: STZ mice treated with CAPA for another 2 weeks; AAR: area at risk; ^∗^: *P  *<* *0.05 versus Con; ^#^: *P  *<* *0.05 versus STZ; *n* = 6–8 per group.
